# Sildenafil Citrate-Restored eNOS and PDE5 Regulation in Sickle Cell Mouse Penis Prevents Priapism Via Control of Oxidative/Nitrosative Stress

**DOI:** 10.1371/journal.pone.0068028

**Published:** 2013-07-02

**Authors:** Trinity J. Bivalacqua, Biljana Musicki, Lewis L. Hsu, Dan E. Berkowitz, Hunter C. Champion, Arthur L. Burnett

**Affiliations:** 1 The James Buchanan Brady Urological Institute, Johns Hopkins Medical Institutions, Baltimore, Maryland, United States of America; 2 Vascular Medicine Branch, National Heart Lung Blood Institute, National Institutes of Health, Bethesda, Maryland, United States of America; 3 Department of Pediatrics, University of Illinois, Chicago, Illinois, United States of America; 4 Department of Anesthesiology and Critical Care Medicine, and Biomedical Engineering, Johns Hopkins Medical Institutions, Baltimore, Maryland, United States of America; 5 Pulmonary, Allergy and Critical Care Medicine, University of Pittsburgh Medical Center, Pittsburgh, Pennsylvania, United States of America; University of Colorado, United States of America

## Abstract

Sildenafil citrate revolutionized the practice of sexual medicine upon its federal regulatory agency approval approximately 15 years ago as the prototypical phosphodiesterase type 5 inhibitor indicated for the treatment of male erectile dysfunction. We now provide scientific support for its alternative use in the management of priapism, a clinical disorder of prolonged and uncontrolled penile erection. Sildenafil administered continuously to sickle cell mice, which show a priapism phenotype, reverses oxidative/nitrosative stress effects in the penis, mainly via reversion of uncoupled endothelial nitric oxide synthase to the functional coupled state of the enzyme, which in turn corrects aberrant signaling and function of the nitric oxide/cyclic GMP/protein kinase G/phosphodiesterase type 5 cascade. Priapism tendencies in these mice are reverted partially toward normal neurostimulated erection frequencies and durations after sildenafil treatment in association with normalized cyclic GMP concentration, protein kinase G activity and phosphodiesterase type 5 activity in the penis. Thus, sildenafil exerts pleiotropic effects in the penis that extend to diverse erection disorders.

## Introduction

Recurrent ischemic priapism, a disorder of non-willful, excessive penile erection, occurs commonly in the sickle cell disease (SCD) patient population and is associated with significant adverse consequences including erectile tissue damage, erectile dysfunction, and psychological distress [1]. The molecular pathophysiology of this erection disorder has only recently been scientifically investigated. Current advances in this field suggest the prominent role of aberrant function of the nitric oxide (NO)-based signal transduction pathway, which is well-described as the main mediatory system for penile erection [2]. Exaggerated erectile responses consistent with priapism are observed in mice lacking the gene for endothelial NO synthase (eNOS), which catalyzes endothelial NO production [3], [4]. Defective phosphodiesterase type 5 (PDE5) regulatory function in the penis accounts for these responses, resulting from altered endothelial NO signaling with detrimental effects on downstream pathway effector molecules, cGMP and cGMP-regulatory protein kinase G (PKG), which regulate PDE5 expression and activity [4], [5]. PDE5 dysregulation also occurs in the penis of a transgenic mouse model of severe sickle cell disease (sickle cell mice) [4], which show a priapism phenotype [6]–[8]. Penile overproduction of adenosine [9], [10] and upregulation of opiorphins (enzymes involved in the polyamine synthesis) [11] in sickle cell mouse penes also may contribute to the priapism phenotype observed in this experimental animal model.

Given the emerging central role of altered NO signaling in the pathophysiology of priapism associated with SCD, we hypothesized that restored normal NO/cGMP/PKG/PDE5-mediated penile vascular homeostasis in the penis via sustained pharmacotherapeutic inhibitory targeting of PDE5 would attenuate this disorder. In uncontrolled clinical studies involving men with SCD, PDE5 inhibitor therapy administered by continuous, long-term dosing unassociated with sexual stimulation proved efficacious in reducing priapism-related events [12], [13]. However, the precise pathogenic mechanisms resulting in altered NO signaling in the penis leading to SCD-associated priapism are unknown. Moreover, the mechanism of action of regimented PDE5 inhibitor therapy as an intervention for priapism has not been fully defined. Therefore, this study was designed, using the sickle cell mouse model, to: 1) investigate the pathogenic mechanisms in the penis causing endothelial NO/cGMP/PKG/PDE5 derangements that predispose priapism, and 2) identify a pharmacologic mechanism by which PDE5 inhibitors potentially serve as a therapy for priapism related to SCD.

## Materials and Methods

### Mouse Model of Human Sickle Cell Disease

Transgenic sickle cell (Sickle) mice with knockout of all mouse hemoglobin genes and expressing exclusively human sickle hemoglobin were developed at Lawrence Berkeley National Laboratory [14]. A breeding colony at the National Institutes of Health (NIH) generated animals for this study by mating sickle male mice to hemizygous females (approximately 15 generations). Because C57BL/6 is one of the background strains for the transgenic sickle mice, C57BL/6 was chosen as WT control. Additional control animals were hemizygous (Hemi) littermates. All were males ages 4 to 6 months old. Mice were pathogen free and received routine NIH rodent chow and water. Studies were approved by the animal care and use committees of the Johns Hopkins Hospital.

### PDE5 Inhibitor Therapy

WT and Hemi mice received oral treatment with the PDE5 inhibitor, sildenafil citrate (Viagra, Pfizer) for 3 weeks, which was provided by mixing drug (100 mg/kg/day) into semi-soft rodent chow (Bioserv; 4−6 g/d) [15], [16]. Mean free plasma concentration of sildenafil was approximately 10–20 nM, a concentration of drug that inhibits 50% of PDE5 activity. This is comparable to levels obtained in humans at doses of 25 mg dosed three times a day and reflects the near 100-fold higher rate of metabolism of sildenafil in the mouse [16].

### Physiologic Erection Studies

*In vivo* erectile function in response to cavernous nerve stimulation (CNS) was studied in anesthetized mice as previously described [6]. Briefly, surgical pelvic dissection was performed for CNS and intracavernous pressure (ICP) monitoring. Each mouse underwent CNS at a frequency of 15 Hz and pulse width of 30 milliseconds. The application of 2 volts was used in the current protocol to achieve a significant and consistent erectile response. The primary *in vivo* erectile function end point to determine the influence of PDE5 inhibitor therapy on prolonged erections observed in Sickle mice was the frequency of spontaneous erections per hour calculated pre and post CNS [6]. Increases in ICP pressure were also measured after direct intracavernous injection of sildenafil citrate (30 nmol/kg) and the NO donor DEA/NO (0.3 µg/kg) [4].

### Collection of Tissue Specimens

Penile specimens were obtained by cutting the crura of the corpus cavernosum at the point of adhesion to the lower pubic bone. Samples were snap frozen in liquid nitrogen, and stored at −80°C until processing for molecular analyses. Molecular analyses were determined under basal (unstimulated) and stimulated conditions. For stimulated conditions, penes were removed during maximal ICP pressures during CNS [4].

### Cyclic Nucleotide Assay

Quantitative assays for cGMP were performed using a commercial enzyme immunoassay kit (Amersham, Piscataway, NJ). For penile cGMP content, frozen penile tissue was homogenized in 6% trichloroacetic acid (1 ml/100 mg tissue), centrifuged, and extracted with water-saturated diethyl ether [4]. cGMP was expressed as fmol/mg protein.

### NOS, PDE5, and PKG Activity Assays

Penile NOS activity was assayed by radiolabelled L-arginine to L-citrulline conversion [4]. Measurements were performed in the presence [for constitutive NOS activity] or absence [for inducible NOS (iNOS) activity] of calcium. Total low K_m_ cGMP PDE5 activity was assayed in duplicate at 1 μM substrate by the 2 step method described previously, under linear conditions with and without added sildenafil citrate (0.1 nM-10 μM) with 0.1 mg/ml BSA and 0.1 mM EGTA [4]. PKG activity was determined by colorimetric analysis (CycLex, Nagano, Japan) according to manufacturer's instructions [5].

### Reactive Oxygen Species (ROS) and Nitrotyrosine Analysis

Superoxide anion production in penile tissue homogenates was determined by luminol-enhanced chemiluminescence (EMD Biosciences). Penile lysate was resuspended in assay buffer to a final concentration of 100 µM luminal as described [15]. Peroxynitrite was quantitatively assessed by the measurement of nitrotyrosine by an ELISA assay (Oxis International) [15]. Dihydroethidine (DHE; Molecular Probes), an oxidative fluorescent dye, was used to evaluate superoxide anion levels *in situ* [15]. DHE fluorescence images were obtained with an upright fluorescence microscope (Nikon). In a separate series of experiments, penile lysate was incubated with a non-selective NOS inhibitor, N(^G^)-nitro-L-arginine methyl ester (L-NAME; 1 mM), oxypurinol (1 mM), or apocynin (10 µM) before determination of ROS production [15].

### eNOS Dimer/monomer Ratio by Western Blot

SDS-resistant eNOS dimers and monomers in penile tissue were assayed using low-temperature SDS-PAGE under nonreducing conditions. eNOS was immunoprecipitated, and resulting samples were added to Tris glycine 6% gels (Invitrogen Corp.). Electrophoresis was performed in an ice bath at 4°C and gels were stained (SimplyBlue; Invitrogen Corp.) and destained with water [17].

### Statistical Analysis

Data were expressed as mean ± S.E.M. Differences between multiple groups were compared by analysis of variance (ANOVA) followed by a Tukey's multiple comparisons test. Two-group analysis was performed by *t*-test (paired or unpaired as appropriate). Serial studies were tested by repeated measures ANOVA. P value of less than 0.05 was used as criteria for statistical significance.

## Results

### Downregulated NO Signal Transduction Pathway in the Sickle Cell Mouse Penis

**Figure 1:**
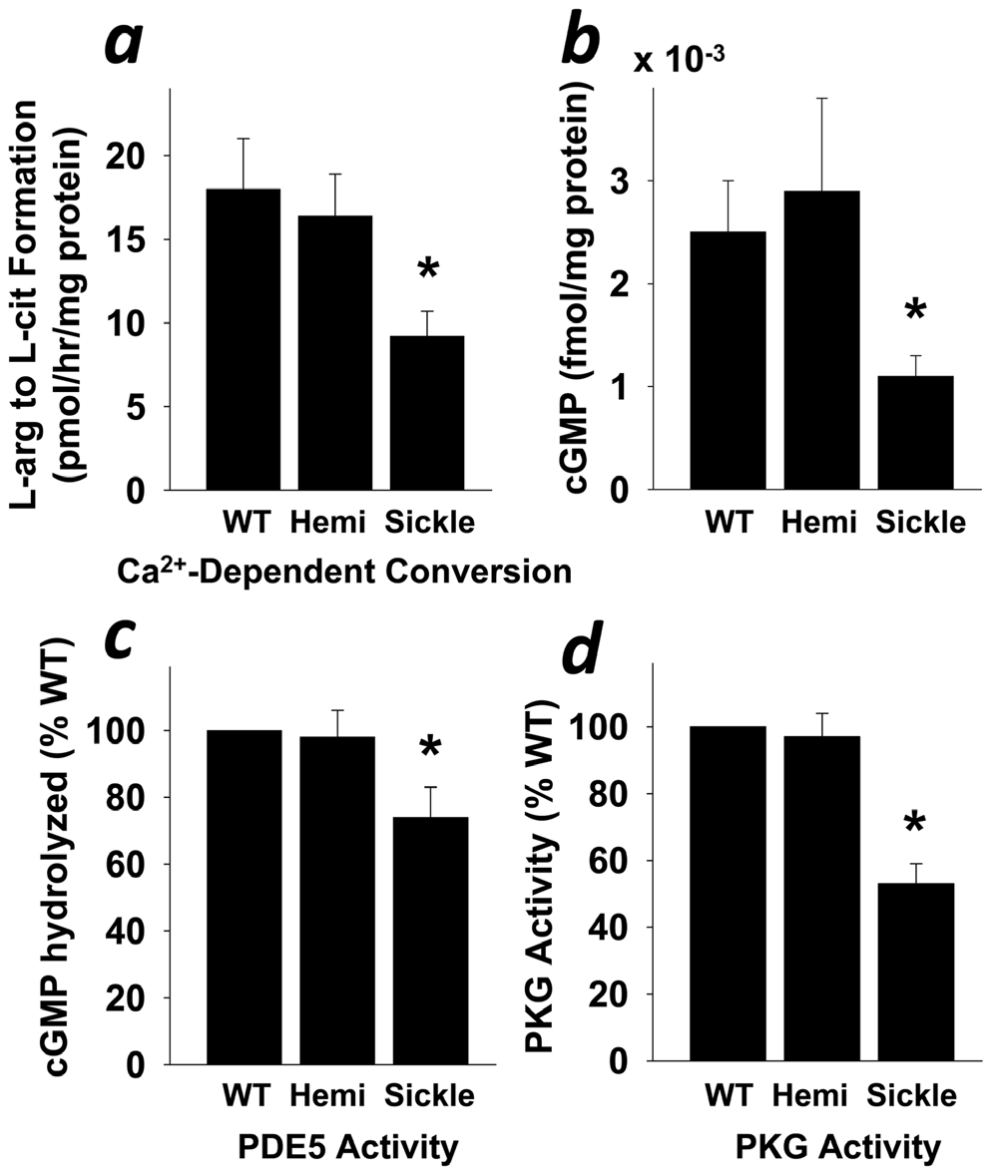
Downregulation of NO/cGMP/PKG pathway in the penis of Sickle mice. Baseline calcium-dependent (constitutive) NOS activity (a), cGMP production (b), PDE5 activity (c), and PKG activity (d) are reduced in the penis of Sickle compared to WT and Hemi mice. Each bar represents the mean ± SEM of 5 mice using ANOVA post-hoc Tukey test. *P<0.05 vs. WT and Hemi.

**Figure 2:**
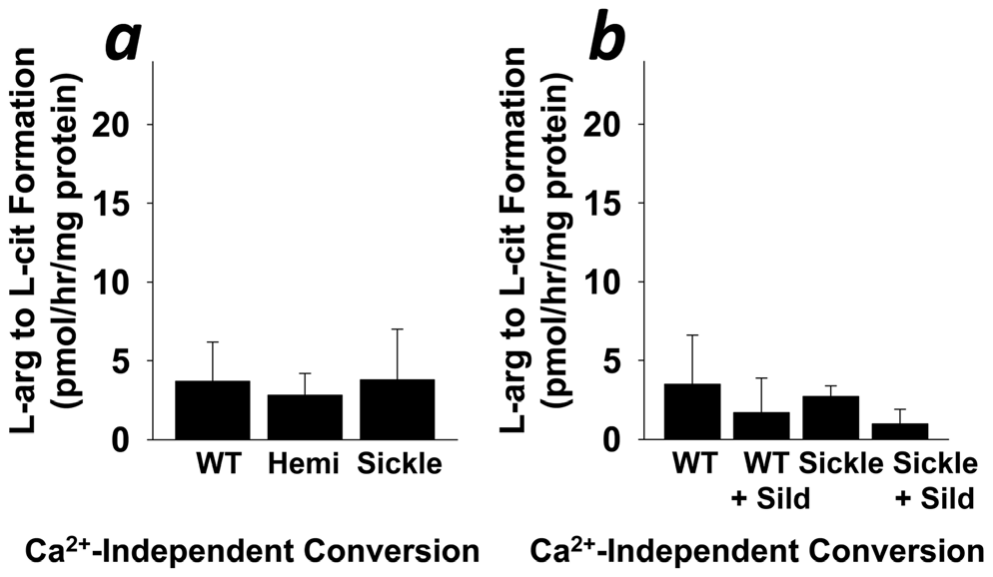
Calcium-independent NOS activity is not affected by SCD (a) or continuous sildenafil treatment (b). Each bar represents the mean ± SEM of 5 mice using ANOVA post-hoc Tukey test.

Advancements in the pathophysiology of priapism suggest that defective neurovascular control of an erection episode accounts for priapism, as a consequence of PDE5 dysregulation resulting directly from altered NO/cGMP/PKG signaling in the penis [4], [5]. In order to confirm our previous observations in eNOS-deficient mice, we and others have shown that sickle cell mice have impaired eNOS protein and cGMP synthesis, which manifests as priapism in vivo and enhanced corporal smooth muscle relaxation in vitro [6], [7], [9], [10], [18]. Herein, we evaluated the regulatory biology of the NO/cGMP/PKG/PDE5 pathway in the sickle cell mouse penis. We found that constitutive (calcium-dependent) NOS activity was reduced (P<0.05) in penes of homozygote (Sickle) compared to hemizygous (Hemi) sickle cell mice and their wild-type (WT) counterparts (Fig. 1a), while there was no difference between groups for penile non-constitutive (calcium-independent) NOS activity (Fig. 2a). We also found that basal cGMP production, PDE5 activity, and PKG activity were reduced (P<0.05) in Sickle compared to Hemi and WT mouse penes (Fig. 1b, c, d). For each of these measurements, the Hemi mice were similar to WT mice. These findings suggest that the tonic synthesis of physiologically relevant NO is reduced, accompanied by a downregulation of its biochemical signaling cascade in the penis in association with SCD.

### Increased Oxidative/Nitrosative Stress in the Sickle Cell Mouse Penis

**Figure 3:**
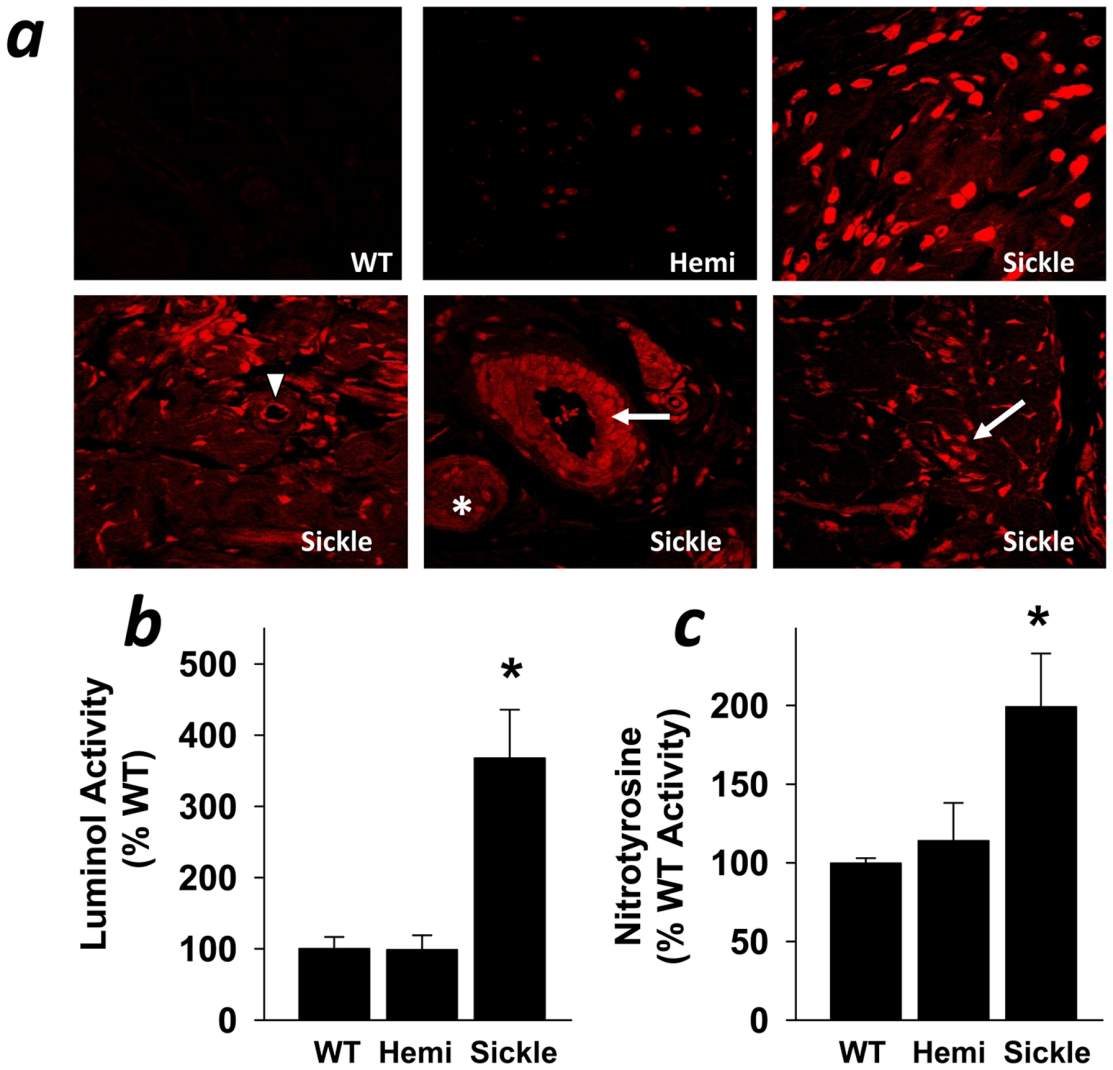
Penes of Sickle mice exhibit increased production of ROS compared to WT and Hemi mice. Superoxide production was measured by DHE fluorescence (a). Arrows indicate smooth muscle cells; arrowheads indicate endothelial cells; asterisks indicate neurons. ROS production was measured by luminol-dependant chemiluminescence (b), and protein tyrosine nitration by nitrotyrosine, a marker of peroxynitrite formation (c). Each bar represents the mean ± SEM of 6 WT and Sickle mice penes samples and 3 Hemi mice penes samples using ANOVA post-hoc Tukey test. *P<0.05 vs. WT and Hemi.

Oxidative and nitrosative stress contribute to the cavernosal tissue damage of priapism in association with reperfusion injury following episodic relief of ischemia in the penis [19]. We wanted to determine what redox-active species are present in the penis in association with SCD. We found that DHE fluorescence, indicating superoxide production, was uniformly increased in sections of the penis from Sickle compared to WT mice (Fig. 3a). DHE localization was increased in all cell types in the Sickle penes, including endothelial and smooth muscle cells as well as penile neurons (Fig. 3a). Similarly, luminol activity, indicating reactive oxygen species (ROS) production, and nitrotyrosine activity, indicating protein tyrosine nitration by peroxynitrite, were increased (P<0.05) in the penis of Sickle compared to Hemi and WT mice (Fig. 3b, c). These results suggest that oxidative stress is increased in the penis in association with SCD.

**Figure 4:**
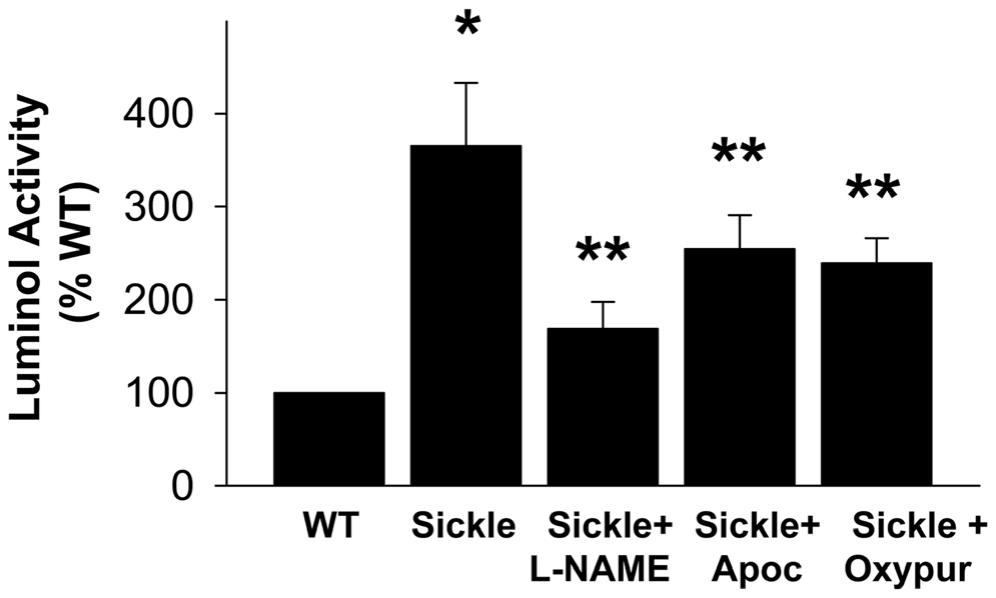
ROS production is decreased in the presence of NOS, NADPH oxidase, and xanthine oxidase inhibitors. All 3 inhibitors (L-NAME 1 mM, apocynin 10 µM, oxypurinol 1 mM, respectively) decrease the production of ROS from penes of Sickle mice, indicating that NOS, NADPH oxidase, and xanthine oxidase are sources of ROS in penes of Sickle mice. ROS production was measured by luminol activity. Each bar represents the mean ± SEM of 6 mice using ANOVA post-hoc Tukey test. *P<0.05 vs. WT; **P<0.05 vs. Sickle.

### Abnormal Erectile Responses of the Sickle Cell Mouse

**Figure 6:**
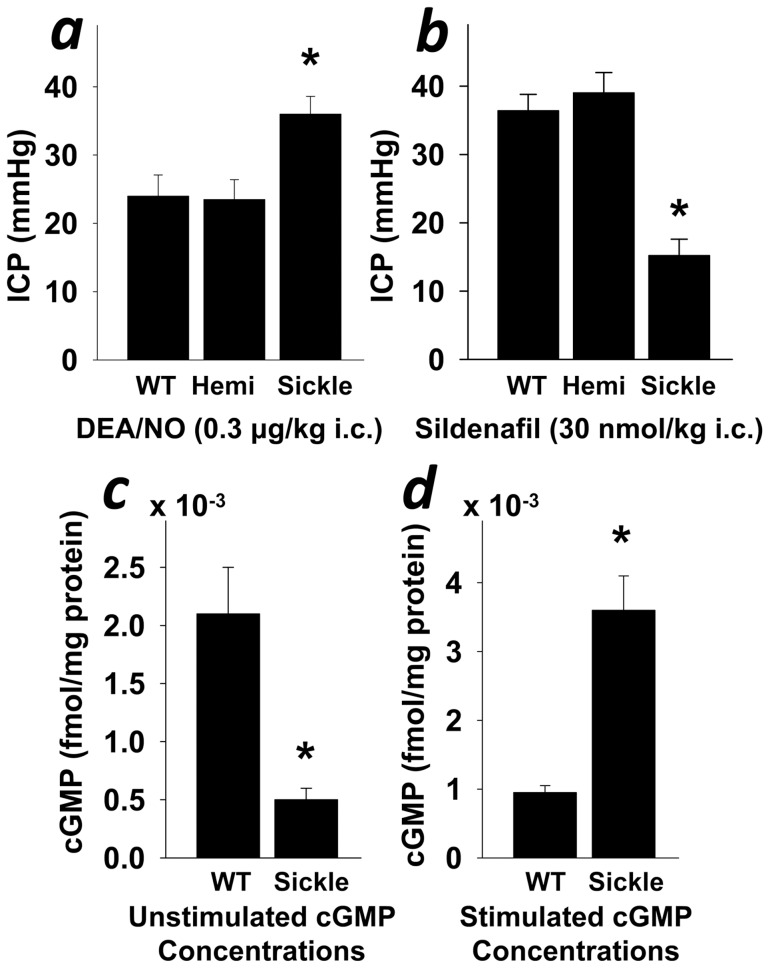
Priapism in Sickle mice is due to unrestrained cGMP/PDE5 function. Intracavernous pressure (ICP) is increased in response to intracavernosal (i.c) injection of the NO donor DEA/NO (0.3 µg/kg) (a) and is decreased in response to i.c. injection of sildenafil citrate (30 nmol/kg) (b) in Sickle compared to WT and Hemi mice. Basal cGMP levels are decreased (c), while neurostimulated cGMP levels are increased (d) in the penis of Sickle compared to WT mice. Each bar represents the mean ± SEM of 8 WT and Sickle mice and 6 Hemi mice; unpaired t-test were used to determine statistical significance. *P<0.05 vs. WT and Hemi.

We have recently demonstrated that sickle cell mice display spontaneously priapic activity and pronounced erectile responses to electrical stimulation of the cavernous nerve, as well as prolonged erections after discontinuation of neurostimulation [6]. To evaluate whether the priapic phenotype is due to unrestrained cGMP levels as a result of PDE5 dysfunction, we conducted erection pharmacostimulation studies in WT, Hemi, and Sickle mice. Separate in vivo experiments using local intracavernous injection of NO donor and a PDE5 inhibitor were conducted. Intracavernous injection of the NO donor DEA/NO increased erection (ICP) in all 3 groups, but the increase was markedly more pronounced (P<0.05) in Sickle mice (Fig. 6a). On the contrary, while intracavernous injection of the PDE5 inhibitor sildenafil citrate also increased ICP in all 3 groups, this increase was markedly less pronounced (P<0.05) in Sickle mice (Fig. 6b). These results suggest that in the Sickle mouse penis the accumulation of the NO effector cGMP is not degraded due to the lack of functional PDE5 activity and there is a lack of basal, inhibitable PDE5 activity in their penes from which to induce erectile responses, respectively. To further prove this conjecture, we measured cGMP levels in the penis at baseline and upon neurostimulation. In contrast to decreased penile cGMP levels at baseline (Fig. 1b and Fig. 6c), cGMP levels were elevated (P<0.05) upon cavernous nerve electrical stimulation in Sickle compared to WT mice (Fig. 6d), indicating unchecked cGMP levels as a basis for priapism in the Sickle mice penes.

### Reversal of Prolonged Erection Tendencies by Continuous Sildenafil Treatment in the Sickle Cell Mouse

**Figure 7:**
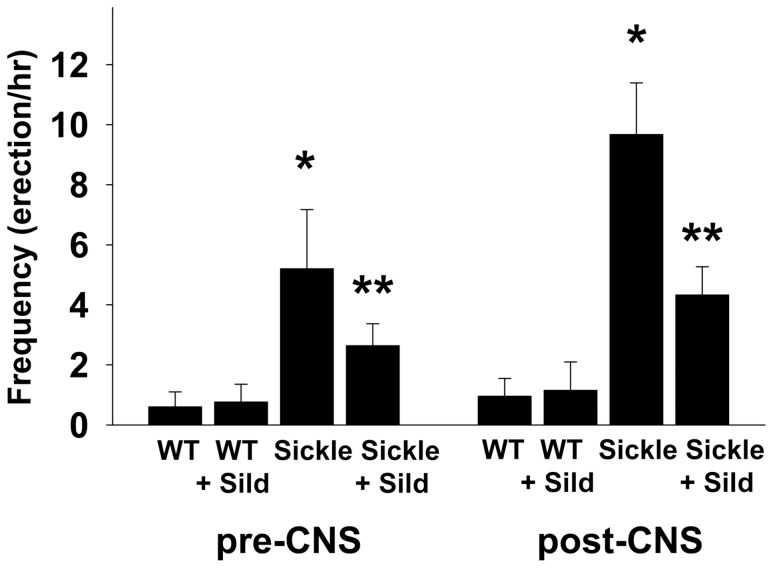
Normalized penile erection by sildenafil treatment. Continuous sildenafil citrate treatment (daily for 3 weeks, 100 mg/kg orally) decreases the frequency of spontaneous erections (erections/hr) before and after cavernous nerve stimulation (CNS; 2 volts) in Sickle mice. Each bar represents the mean ± SEM of 6 WT and WT+sildenafil and 7 Sickle and Sickle+sildenafil treated mice; ANOVA post-hoc Tukey test and repeated measures of ANOVA were used to obtain statistical significance. *P<0.05 vs. WT, **P<0.05 vs. Sickle.

We have made clinical observations that continuous, long-term PDE5 inhibitor administration to patients with recurrent ischemic priapism results in alleviation or resolution of priapism recurrences [12], [13]. To explore whether the functional effects of this intervention are replicated in the animal model, we treated Sickle mice and their WT counterparts with daily dosing of the PDE5 inhibitor prototype sildenafil for 3 weeks. We found that daily sildenafil administration to Sickle mice decreased (P<0.05) frequency of erectile responses pre- and post-cavernous nerve electrical stimulation (Fig. 7) and decreased (P<0.05) the detumescence phase of erection (area under the erectile curve once the stimulation was terminated: 11.1±2.1 mmHg•sec in Sickle mice treated with sildenafil vs 9.0±2.6 mmHg•sec and 19.7±1.2 mmHg•sec in WT and Sickle mice, respectively. Daily PDE5 inhibitor therapy had no effect on frequency of erections pre- and post-neurostimulation in WT mice. These results suggest that sildenafil administered according to a daily treatment regimen in the setting of SCD-associated priapism effectively reverts abnormal erectile mechanisms toward normal penile vascular homeostasis in the penis.

### Normalized NO Signal Transduction Pathway Function in the Sickle Mouse Penis by Continuous Sildenafil Treatment

**Figure 8:**
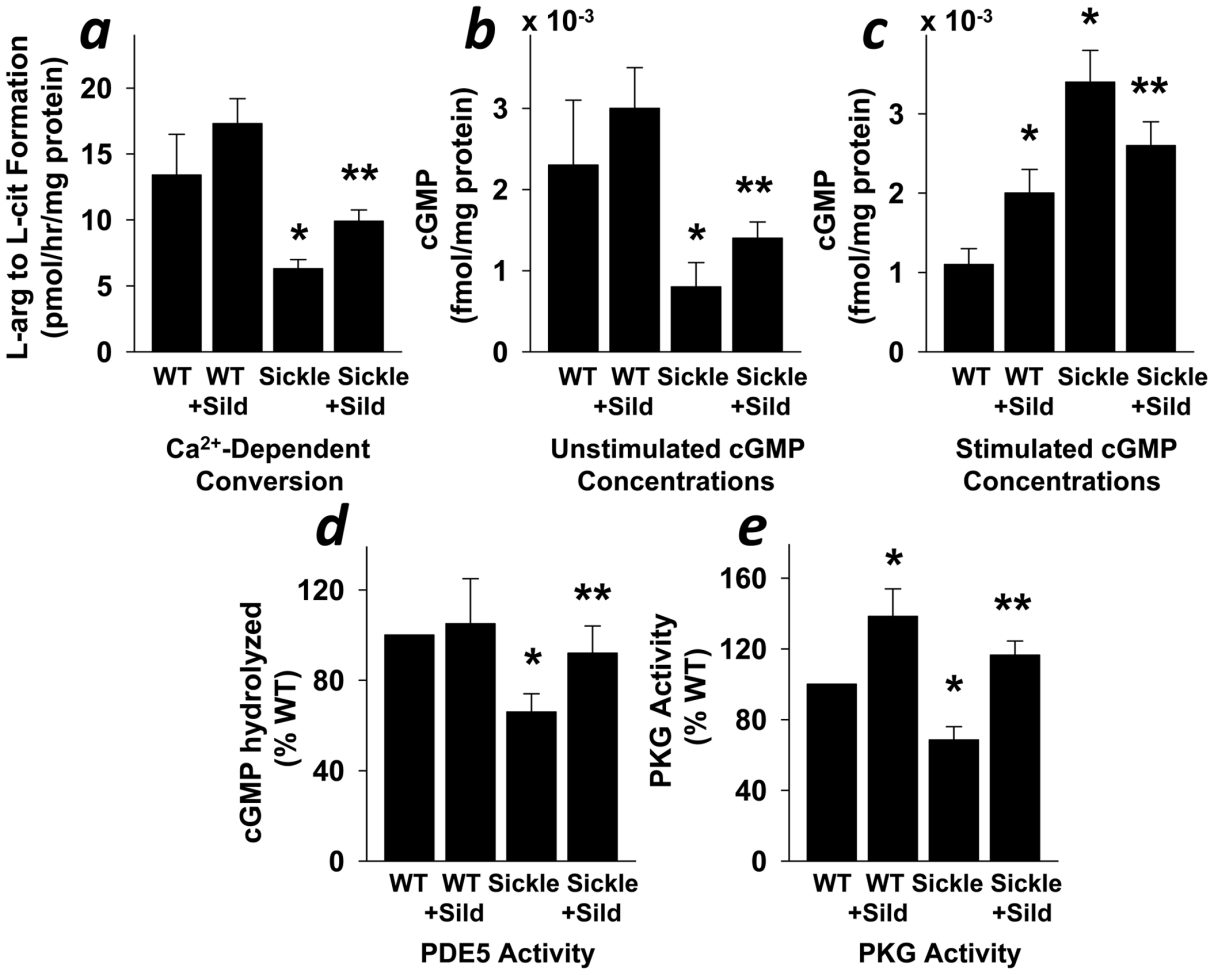
Normalized NO signal transduction pathway in the penis of Sickle mice by sildenafil treatment. Continuous sildenafil citrate treatment increases basal calcium-dependent NOS activity (a; n = 6), cGMP levels (b; n = 7), PDE5 activity (d; n = 6), and PKG activity (e; n = 6), and decreases neurostimulated cGMP levels (c; n = 7) in the penis of Sickle mice. Each bar represents the mean ± SEM using ANOVA post-hoc Tukey test. *P<0.05 vs. WT, **P<0.05 vs. Sickle.

In the current series of experiments, we evaluated the possible mechanisms whereby daily administered sildenafil influences priapic activity associated with SCD in this experimental animal model. We evaluated functional roles of molecular components of the NO signal transduction pathway in penile tissue of Sickle and WT mice in response to daily sildenafil treatment. We found that the basally low penile calcium-dependent NOS activity, cGMP concentrations, PDE5 activity, and PKG activity were upregulated (P<0.05) toward normal levels in Sickle mice after continuous sildenafil treatment (Fig. 8a, b, d, e). Continuous sildenafil treatment also decreased (P<0.05) abnormally high levels of cGMP in the penis of Sickle mice upon neurostimulation (Fig. 8c). There was no change in non-constitutive NOS activity at baseline in WT and Sickle mice penes after therapy (Fig. 2b). These results suggest that the molecular basis for restoration of erection physiology associated with sildenafil treatment in Sickle mice relates to a normalization of molecular derangements in the NO/cGMP/PKG/PDE5 signaling pathway in the penis. The stimulatory effect (P<0.05) of continuous sildenafil treatment on PKG activity and cGMP levels in the penis of WT mice upon neurostimulation (Fig. 8c, e) is conceivably due to overinhibition of PDE5 in the penis of healthy mice.

### Reduced Oxidative/Nitrosative Stress in the Sickle Mouse Penis with Continuous Sildenafil Treatment

**Figure 5:**
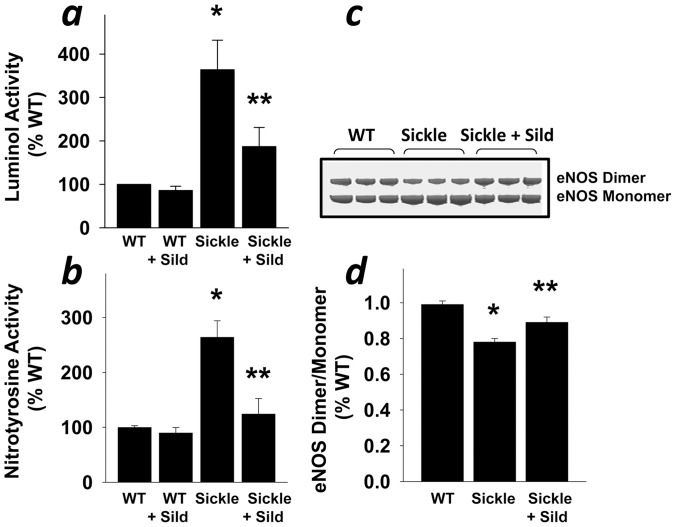
Reduced ROS production and eNOS uncoupling in the penis of Sickle mice by sildenafil treatment. ROS production was measured by luminol activity (a; n = 4–6) and nitrotyrosine activity (b; n = 7). Representative Western blots (c) and densitometric analysis (d; n = 5) of eNOS dimers and monomers in the penis after low-temperature SDS-PAGE. eNOS is uncoupled in the penis of Sickle mice compared to WT mice, while continuous treatment of Sickle mice with sildenafil citrate recouples eNOS. The results are expressed as dimer/monomer ratio. Each bar represents the mean ± SEM using ANOVA post-hoc Tukey test. *P<0.05 vs. WT; **P<0.05 vs. Sickle.

Sildenafil reduces oxidative stress in the peripheral and penile vasculature with associated improvement in endothelial NO function [15], [20], [21]. Therefore, we hypothesized that continuously administered sildenafil may exert benefits by influencing oxidative/nitrosative stress and endothelial cell biology in the penis under conditions predisposing priapism. We found a reduction (P<0.05) in luminol chemiluminescence and nitrotyrosine activity in the penis of Sickle mice in response to daily sildenafil treatment (Fig. 5a, b). We also found that this treatment increased (P<0.05) the eNOS dimer/monomer ratio in penes of Sickle mice, suggesting that it prevented eNOS uncoupling (Fig. 5c, d). These results suggest that daily administered sildenafil increased eNOS activity by preventing oxidative stress and particularly eNOS uncoupling in the Sickle mouse penis, which restored normal erection physiology as it reduced the priapism phenotype.

## Discussion

In the present study, we demonstrate that sildenafil reverses aberrant signaling of the NO/cGMP/PKG/PDE5 axis that accounts for abnormal neurovascular control of erectile function in vivo in an animal model of SCD. The priapic activity that is observed in the sickle cell mouse is associated with a reduction in PDE5 regulatory function in the penis due to altered signaling of endothelial NO and its downstream effectors, cGMP and PKG. The molecular mechanism underlying the reduction in constitutive NOS activity involves elevated oxidative/nitrosative stress, principally as a result of functional uncoupling of eNOS. As a result of PDE5 dysregulation, cGMP generated in abundance in the erectile tissue during a finite episode of neurostimulation cannot be degraded and produces unrestrained corporal tissue relaxation resulting in priapism. This study further establishes that continuous PDE5 inhibitor therapy restores normal cGMP/PKG/PDE5 functional levels in erectile tissue allowing proper degradation of episodically generated cGMP, consistent with controlled corporal vasorelaxation and erectile function. This action of sildenafil involves control of oxidative stress principally via reversion of uncoupled eNOS to functional coupled eNOS in the penile vasculature, thus restoring endothelial NO biosynthesis and its downstream signaling.

An important mechanism of decreased endothelial NO bioavailability is functional uncoupling of eNOS, characterized by the diversion of electron transfer within the enzyme from L-arginine oxidation. This molecular event reduces molecular oxygen to form superoxide instead of NO [22]. Uncoupled eNOS, thus, contributes to decreased NO bioavailability and increased ROS formation by the enzyme, conditions which are both prevalent in SCD-associated vasculopathy [17], [23]–[25]. Uncoupled NOS appears to provide a dominant source of ROS in the penis in SCD, because the NOS inhibitor L-NAME reduced luminol chemiluminescence more than 50% in the sickle cell mouse penis. Furthermore, the ratio of functional eNOS dimers to nonfunctional monomers was decreased in the penis of sickle cell mice, implying eNOS uncoupling with the loss of its dimerization. The precise mechanism by which eNOS becomes uncoupled is unclear at this time but may be related to oxidation of the critical NOS cofactor BH_4_, depletion of L-arginine, accumulation of endogenous methylarginines, or S-glutathionylation of eNOS [26]. NADPH oxidase and xanthine oxidase also contribute towards oxidative stress in the sickle cell mouse penis. However, the contributions of NADPH oxidase and xanthine oxidase are apparently smaller compared to that of uncoupled eNOS, because inhibition of these enzymes reduced luminol activity more modestly compared to NOS inhibition. Our findings confirm recent studies, which demonstrated that increased oxidative stress generated by NADPH oxidase and uncoupled eNOS in the sickle cell mouse penis contributes to endothelial dysfunction and cavernosal tissue damage [24], [25]. Further reaction of NO with superoxide derived from uncoupled eNOS, xanthine oxidase, or NADPH oxidase produces peroxynitrite, as evidenced by increased levels of protein tyrosine nitration in the sickle cell mouse penis. Peroxynitrite is an extremely toxic reactive nitrogen species, which may further uncouple eNOS. Non-constitutive iNOS can also contribute to oxidative stress in pathological conditions by producing excessive NO, which reacts more rapidly with superoxide to produce peroxynitrite [27]. However, iNOS does not appear to be an important source of ROS in the sickle cell mouse penis as calcium-independent NOS activity was low and not altered in this animal model.

Both increased oxidative stress and eNOS uncoupling decrease endothelial NO bioavailability in the penis in association with SCD. Endothelial NO bioavailability in the sickle cell mouse penis is also reduced by decreased eNOS phosphorylation on its positive regulatory site Ser-1177 and by decreased eNOS- heat shock protein (HSP) 90 interaction, as we recently reported [18]. Under reduced endothelial NO bioactivity at basal, steady state conditions, cGMP is produced only in low amounts, which is insufficient to maintain the proper set point for PDE5 function due to lack of the cGMP-dependent feedback control mechanism [12], [28]. The notion that PDE5 is downregulated is further supported by the increased erection physiologic response of sickle cell mice to intracavernously administered NO donor (because NO effector cGMP cannot be degraded), and their decreased erectile responses to intracavernously administered sildenafil (because of the lack of inhibitable PDE5 activity necessary to increase cGMP). Our in vivo findings confirm previous in vitro findings of downregulated PDE5 in cavernosal smooth muscle cells of rats [28], mice [4], and humans [29] under hypoxic conditions, which mimic the penile ischemia of priapism. These data suggest that the set point of PDE5 activity in the penis corresponds with endothelial NO bioavailability in this organ providing new insight into the regulatory basis for functional penile vascular homeostasis as it relates to priapism.

Over the past several years PDE5 inhibitors have been explored for their beneficial therapeutic effects beyond treating male ED. In the peripheral vasculature [21], [30], [31] as well as in the penis [15], [32], [33], PDE5 inhibitors exert beneficial effects on eNOS function and vascular oxidative stress. We now show that continuous treatment with sildenafil reduced oxidative stress and prevented eNOS uncoupling in the sickle cell mouse penis. By recoupling eNOS, sildenafil both decreases ROS production and increases NO production by the enzyme. While the mechanism underlying this effect of sildenafil on eNOS is not known, our data support the hypothesis that it promotes cGMP/PKG-mediated increases in shear stress and consequent stabilization of eNOS coupled function. Functional eNOS replenishes endothelial NO biosynthesis in the penis. This event directly results in improvements in cGMP concentrations and PKG activity in the penile vascular bed, which normatively resets PDE5 enzyme activity. Another possible mechanism involves the ability of PDE5 inhibitors to increase NO biosynthesis and downstream PKG phosphorylation. Increased PKG activity/phosphorylation in turn induces positive feedback conformational changes, increasing cGMP-binding affinity in the regulatory GAF domain of the PDE5 enzyme and enhancing its cGMP catalytic activity. By whichever mechanism, we have shown here that continuous use of a PDE5 inhibitor in a priapism model recovers PDE5 enzymatic activity in the penis.

In conclusion, this study suggests that focus should be given to PDE5 as a molecular target for treating recurrent priapism associated with SCD whereby it directs the use of rational, disease-specific pharmacotherapy for an ill-defined disorder. Importantly, our findings suggest that continuous, long-term treatment with PDE5 inhibitors reverses eNOS uncoupling in the sickle cell mouse penis which restores endothelial NO synthesis, in support of the proposal that “NO imbalance” in the penis is a molecular pathogenic basis for priapism. These observations reveal the pleiotropic effects of PDE5 inhibitors for erection disorders: whereas these drugs are commonly used as erectogenic agents for the treatment of male erectile dysfunction, they also appear useful as regulators of penile vascular homeostasis, beneficial for managing priapism associated with SCD.
